# Faith and HIV prevention: the conceptual framing of HIV prevention among Pentecostal Batswana teenagers

**DOI:** 10.1186/1471-2458-14-225

**Published:** 2014-03-05

**Authors:** Elias Mpofu, Fidelis Nkomazana, Jabulani A Muchado, Lovemore Togarasei, Jeffrey Bart Bingenheimer

**Affiliations:** 1 University of Sydney, Sydney, Australia; 2 University of Botswana, Gaborone, Botswana; 3 George Washington University, Washington, DC, USA; 4 Faculty of Health Sciences, University of Sydney-Cumberland Campus, Room T-428, 75 East Street, Lidcombe, NSW 2141, Australia

**Keywords:** Faith concepts, HIV prevention, Pentecostal, Church, Religion

## Abstract

**Background:**

There is a huge interest by faith-based organizations (FBOs) in sub-Saharan Africa and elsewhere in HIV prevention interventions that build on the religious aspects of being. Successful partnerships between the public health services and FBOs will require a better understanding of the conceptual framing of HIV prevention by FBOS to access for prevention intervention, those concepts the churches of various denominations and their members would support or endorse. This study investigated the conceptual framing of HIV prevention among church youths in Botswana; - a country with one of the highest HIV prevalence in the world.

**Method:**

Participants were 213 Pentecostal church members (67% female; age range 12 to 23 years; median age = 19 years). We engaged the participants in a mixed-method inductive process to collect data on their implicit framing of HIV prevention concepts, taking into account the centrality of religion concepts to them and the moderating influences of age, gender and sexual experience. After, we analysed the data using multi-dimensional scaling (MDS) and hierarchical cluster analysis (HCA) to map the ways the church youths framed HIV prevention.

**Results:**

The findings suggest the church youth to conceptually frame their HIV prevention from both faith-oriented and secular-oriented perspectives, while prioritizing the faith-oriented concepts based on biblical teachings and future focus. In their secular-oriented framing of HIV prevention, the church youths endorsed the importance to learn the facts about HIV and AIDS, understanding of community norms that increased risk for HIV and prevention education. However, components of secular-oriented framing of HIV prevention concepts were comparatively less was well differentiated among the youths than with faith-oriented framing, suggesting latent influences of the church knowledge environment to undervalue secular oriented concepts. Older and sexually experienced church youths in their framing of HIV prevention valued future focus and prevention education less than contrasting peer cohorts, suggesting their greater relative risk for HIV infection.

**Conclusion:**

A prospective HIV prevention intervention with the Pentecostal church youths would combine both faith and secular informed concepts. It also would need to take into account the ways in which these youth interpret secular-oriented health concepts in the context of their religious beliefs.

## Background

In sub-Saharan Africa, churches are among the most important institutions in many communities [1-4]. Their work in providing pastoral care in the context of the region’s HIV epidemics is widely recognized [5-7]. They are also well-positioned to make important contributions to HIV prevention. Churches in sub-Saharan Africa are more trusted by the indigenous population that many secular organizations [8]). Moreover, FBOs have large memberships and a well-developed communications infrastructure, and hence the capacity to disseminate HIV and AIDS education messages [5,9-11]. For these reasons, public health programs in sub-Saharan Africa have increasingly looked to partner with churches in HIV prevention efforts [12,13]. In order to maximize the effectiveness of church-based HIV prevention efforts in sub-Saharan Africa, studies are needed on the ways in which churches understand and implement HIV prevention messages with their congregates.

HIV prevention messages seek to increase knowledge, beliefs, and attitudes that support protective behaviors, and to decrease misinformation and attitudes that promote risky behaviors. Faith-oriented concepts of sexual abstinence until marriage, and monogamous marriage with sexual fidelity relate closely to the first two components of the ABC approach to HIV prevention. Secular teachings, in contrast, would give more emphasis to condom use, life skills education [14] , and to the influences of social norms on sexual behaviours [15,16]. Our study sought to investigate the conceptual framing of HIV prevention by youth who are members of Pentecostal churches in Botswana, and also to characterise the importance that the church youths accorded to the faith-oriented and secular-oriented HIV prevention concepts they held.

### Pentecostal churches in Botswana

Botswana is a landlocked country in southern Africa, bordered by Angola, Namibia, South Africa, Zambia, and Zimbabwe. With an estimated 20 percent of the total population (or 400,000 of 2 million) HIV positive, it has one of the highest HIV infection rates in the world. The prevalence of HIV is 24% among 15–19 year olds and close to 44% among 20–24 year olds [17,18].

According to the Botswana Council of Churches [19], Pentecostal churches comprise 73% of faith-based organizations (FBOs) in the country, and are the majority faith community. These churches emphasize the importance of being born again, being filled with the Holy Spirit, living a holy life, and being prosperous [20,21]. They tend to follow a prosperity view of God as the ultimate gracious benefactor who rewards and forgives those who are faithful in Him [22,23].

Approximately 80% of young people in Botswana are affiliated with Pentecostal churches [19]. These churches aspire to provide HIV education to their youth members. Historically, Pentecostal churches in Botswana have emphasized abstinence until marriage (A) and sexual fidelity within marriage (B) as strategies to avoid contracting HIV [24,25]. Recently, however, and in part as a result of public health education on HIV, many churches in Botswana are increasingly open to a broader set of health protection teachings [26]. For instance, a substantial minority (27%) of Pentecostal churches endorse interventions that promote the use of condoms (C) for preventing HIV transmission [26]. The ways in which Pentecostal church youth understand risk for HIV and strategies for prevention in the context of faith-based and secular messages and broader community norms remains poorly understood [6,27,28].

### Church knowledge environments effects on congregates

Churches are epistemic or knowledge generating and validating environments [29,30]. As such, they transmit to their members encoded expectations about what constitutes valid knowledge and how that knowledge should be acted upon. Many churches prioritize faith-informed over secular ways of knowing. They therefore communicate to their congregations messages about the obligation to adhere to practices and behaviors that are consistent with their core values. When it comes to HIV education, churches may emphasize information and prevention strategies that reinforce faith teachings; and de-emphasize or even condemn information and prevention strategies that contradict established church teachings.

Nevertheless, churches are not the only source of information about HIV for young people. The Botswana government provides 10 years of compulsory basic education, and the school curriculum includes lessons on HIV prevention [31]. Thus, older youths would have been exposed to HIV prevention concepts in-school. Additionally, numerous public education campaigns about HIV have been conducted in Botswana by the government and secular NGOs. These campaigns have provided basic information about HIV and in some cases promoted HIV prevention strategies, including condom use that may be at odds with some church teachings. On the one hand, if FBOs overemphasize abstinence only messages, they may inadvertently encourage disconnect between what youth members publicly profess and the actual private behaviors. On the other hand, in professing a view of God as compassionate and forgiving, the FBOs teachings may be (mis) perceived by some youth as allowing for sexual permissiveness [32]. Young people may also be influenced by social norms related to sexual behaviors that prevail (or that they perceive to occur) among their peers [11,14,15]. The ways, in which young people integrate messages from multiple, possibly conflicting sources may depend upon personal or social characteristics including but not limited to their church affiliation or religiosity.

There presently is little evidence on how Pentecostal church youths frame their HIV prevention concepts in the context of both church and secular community influences. A better understanding of these issues would be important for the packaging of public health oriented messages to reduce risk for HIV with the church teenagers.

### Goals of the study

This study applied state-of-the art concept mapping approaches to gain a better understanding of the ways in which youth in Botswana who are affiliated with Pentecostal churches think HIV prevention. Concept mapping is a mixed-method approach for describing social reality from the view point of the participants, and useful for studying a variety of human service program outcomes [33]. In using concept mapping, participants brainstorm ideas on a concept and they analyze their self-statements in a sequential process that ends with interpretation of the core components and content of the concept (as described below).

Churches may influence what youth members believe about HIV and what strategies for avoiding HIV they see as desirable or valid [29,30]. We hypothesized that the Pentecostal church youth would endorse to understand HIV prevention from both faith-oriented and secular-oriented conceptual frameworks.

We also examined the impact of age, gender, and sexual experience on the salience that Pentecostal youth in Botswana assign to their framing of HIV prevention. For instance, younger teens less focused on sexuality and romantic relationships may adhere more strictly to church teachings about sexuality in their conceptual framing of HIV prevention. In contrast, older youth who are unmarried but sexually active may prioritize secular teachings about HIV, and temper faith-oriented messages to align with their sexual practices (e.g., “It’s okay to have sex with love”.; “To sin is human”.; “God will forgive.”). Such personal framing of sexuality in the context of religion may be facilitated by the view of God as compassionate, loving, and forgiving [32]. Teenage girls in Botswana initiate sexual activity at younger ages than their male peers, on average, often through liaisons with older men [31], and for this reason are at higher risk for HIV infection [3]. We hypothesized that female, older, and sexually experienced youth would attach more importance to secular-oriented framing of HIV prevention, respectively, than would male, younger and sexually inexperienced youth.

The current study makes several contributions to our understanding of HIV prevention with church youths. First, no previous study has mapped implicit HIV prevention concepts held by Pentecotsal church youths for HIV prevention intervention design. In most prior studies, the youths respond to HIV prevention concepts preselected by the investigators. This has the potential limitation to mis-specify the conceptual frameworks that the youths themselves hold to be important and likely to influence their sexual behaviour. This study, in which the church youths themselves explicate their understanding of HIV prevention, has the unique strength to inform an appropriately targeted HIV prevention intervention with them. Second, the factors that influence the salience of specific conceptual framings for HIV prevention with the church youths are not extensively known [33,34], except for the abstinence –until-marriage only concepts [11,23,25]. This is the first study to systematically examine the relative weighting of faith-oriented and secular-oriented conceptual framing of HIV prevention by Pentecostal faith community youths for prospective prevention intervention design with their church organization. In summary, we used a prospective concept mapping approach to profile HIV prevention concepts held by the church youths as influenced by their religion, developmental age, gender and sexual experience.

## Method

### Participants and setting

Our study was conducted with a Pentecostal church that boasts 26 congregations across the country, most of them in urban areas. Teenagers and young adults comprise a large proportion of members. We stratified the congregations according to size and community type and randomly sampled eight: two large (over 250 member), four medium (between 51 and 249 members), and two small (up to 50 members), evenly balanced between rural and urban location. All unmarried youth members of sampled congregations between ages 12 through 23 were invited to participate in the study. We enrolled 213 (female = 67%; age range 12 to 23 years; median age = 19 years) (see Table 1).

**Table 1:** Participant characteristics (N =213)

**Demographic**		**First sex**		
		**Yes (n = 61)**	**No (n = 152)**	
Gender				Totals
	Male	16 (26)	53(34))	69(32)
	Female	45 (74)	100 (66)	145 (68)
Age				
	Younger (<18 years)	4(6)	55(36)	59(29)
	Older (18 years and above)	57(94)	97(64)	154(72)

Note. The numbers in brackets are column percentages with and without first sex.

The higher female enrollment suggests that HIV prevention concepts held by females youths are likely better estimated by this study than those held by their male peers. However, the comparatively higher participation of females in church is typical of FBOs in sub-Saharan African region in which females attend and retain church more than males; -even though the church leadership is mostly male [2-5].

### Procedure and data collection

Permission for the study was granted by the Human Research Ethics review boards of the University of Sydney and the University of Botswana. Participants who were young adults provided written consent to take part in the study. Minor age teenagers (< 16 years) provided written assent for the study with passive parental consent. The minor age teenagers carried home from the church service center a letter seeking consent from the parents one week prior to the data collection. The letters were served in both English and Setswana (the local language). The letter explained the purpose of the study and the requirements of participants, and asked parents to send back a signed form if they did not want their child to participate in the study. At the time of data collection, minor age teenagers whose parents consented permission to participate provided written assent to participate.

Data collection activities were conducted by the lead author and in-country co-investigators at church centers during times mutually agreed upon by the research team and the church organization. Participants used self-selected three letter identification codes known only to themselves for all their data submissions (as described below). The submission of data by participants to the research team did not carry information traceable to a particular participant. This enhanced the chances that participants would be frank about their observations.

### Statement brainstorming workshops

We hosted two half-day workshops with youth from each congregation. The first workshop was devoted to brainstorming. The research team provided an introduction in which we informed participants that we wanted to learn about their HIV prevention concepts. Next we asked participants to complete and submit in sealed envelopes a demographic information sheet covering gender, age, and whether each participant had ever had sex.

Participants were then given a form individually for recording the ways in which they understood HIV prevention. This form included written instructions, and there were four distinct versions of these instructions. All four versions included the following text: “Think about an HIV/AIDS prevention curriculum that would be helpful to teenagers in your church that prevent them from contracting HIV. As you think about these issues, please generate as many statements (short phrases or sentences) as you can and list them below”. But depending upon their response on the demographic data sheet, some solicitation instructions began with the statement, “You have engaged in sexual intercourse before”, whereas others began with, “You have not engaged in sexual intercourse before”. The instructions on approximately half of the forms (determined by split-ballot) ended with the phrase, “Based on my faith or beliefs, a HIV prevention curriculum for teenagers in my church should include….” Those on the other half ended with the phrase, “Based on my personal beliefs, a HIV prevention curriculum for teenagers in my church should include….” Thus, the intersection of sexual experience by probe type determined the overall wording of the HIV prevention concept solicitation (see Table 2). The participants were invited to write their statements in either English or Setswana.Our use of two versions of the probe, on explicitly faith-oriented and one neutral, was based upon the premise that socio-behavioral thoughts can be reliably primed to reveal implicitly held beliefs [35,36], including in religious situations (see also [37] for a review).

**Table 2:** Number of participants who took the faith belief or personal belief statement probe for the brainstorming and self-reporting on first sex (N = 213)

**First sex**	**Statement probe condition**	
	**A. Faith belief**	**B(Personal belief)**
C = Yes (n = 61)	28 (25)	33 (32)
D = No (n = 152)	82(75)	76(68)
Totals	110 (51)	103(49)

Note. The numbers in brackets are column percentages. AC = number of participants with first who took the faith oriented statement probe self-reporting with first sex. BC = number of who took the faith oriented statement probe self-reporting with first sex; AD = number of participants who took the personal belief statement probe self-reporting with no first sex; BD = number of participants who took the personal belief statement probe self-reporting with no first sex.

Finally, we engaged participants in whole-group discussions to clarify the meanings of some of the submitted statements (with no attribution). We informed participants that all free-listed statements were admissible and that, in group discussion, criticism of others was to be avoided.

### Statement content auditing

Upon completion of the eight statement solicitation and clarification workshops, three members of the project team content edited the statements for duplications and produced a final list of 50 unique statements. We also individually content analyzed the statements for possible content biases, contrasting the statements from the youth who received the faith-based probe with those from youth who received the neutral probe. We observed a mean Kendall tau concordance of agreement = .93 among the three raters, suggesting that the youths HIV prevention concepts were similar, regardless of the statement probe they responded. We placed each of the statements on a 3” × 5“card with duplication so each of the participants had a full deck of 50 cards for the sorting and rating workshop.

### Statement sorting and rating workshops

We next held a second workshop with participants from each of the eight congregations. At these workshops we conducted sorting (personal framing) and importance rating (salience) activities. In the sorting activity, each participant was provided with a bundle of 50 cards with numbered individual statements from the brainstorm session and asked to group the statements into piles “in a way that make sense to you”. We asked participants to write the number for each statement on a record sheet so that statements grouped together were in the same cluster. Participants provided a short descriptive label for each of the clusters to capture their perceived core meaning for the cluster.

For the salience rating task, participants used a listing of the 50 statements in questionnaire format and to rate each of the statements for relative importance to their HIV prevention on a 5-point Likert-type scale (*relatively unimportant = 1, to extremely important = 5*). We also asked participants to self-report demographic information (e.g., sexual experience, age, gender) on an information blank attached to the statement sorting and rating forms.

### Data analysis

For the analysis, we used Concept Systems software [38] to model the representation of HIV risk prevention by the church youths in the context of their religious organization. At least 11 respondents per participant grouping by demographics are required for concept systems maps [33]. Our sample size by church youth demographics (other than younger youths with no first sex) far exceeded the minimum required - adding to confidence in the reliability of the data for the analysis. We describe the specific analytical procedures aligned with our research aims as previously stated.

### Structural and salience analysis

**Figure 1:**
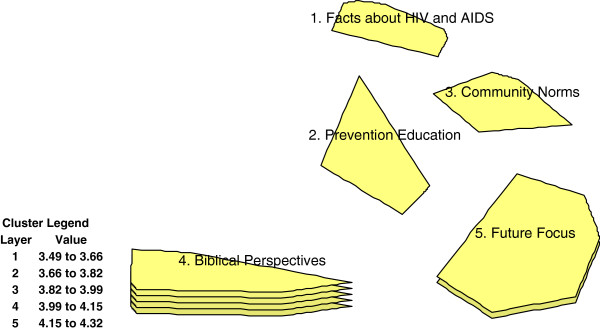
**Cluster rating map for messages perceived to be received by Botswana Pentecostal youths orphan to influenced their sexual decisions.** Participants rated message statements on the criterion of importance to their HIV prevention decisions. Higher stacked clusters indicate those considered particularly important to the youth HIV prevention. With an observed sten statistic of .27, the five cluster solution explains 73% the variance in the data; - suggesting a high stability or repeatability with a similar sample of church youths.

The Concept Systems program utilizes multidimensional scaling (MDS) and Hierarchical Clustering analysis (HCA) to construct concept maps from the participants’ sorting of the statements. In applying MDS with free listed statements data, the Concept System software locates statements judged to be similar by the participants more proximately than those that are piled together less frequently. In addition, Concept Systems then applies HCA to overlay the importance ratings (1–5) onto the proximity configuration from the MDS (as previously described). The *cluster rating map* (as in Figure 1 below) is the final product, showing the bounded item groupings with importance ratings stacking. Concepts with higher salience or importance show with thicker or multi-layered clusters.

Concept Systems generates a *sten statistic* to measure the stability of the cluster solutions. It is interpreted like Wilk’s Lambda (λ) in that lower indicator values denote higher accounted-for variance from the specified cluster solution, hence its reproducibility.

### Contrastive analysis for effects of, gender, sexual experience and age

We computed Welch-Aspin unpaired t-tests to contrast the relative salience of HIV prevention concept clusters to the Pentecostal youths by their sexual experience, age, and gender. The Welch-Aspin index is appropriate with samples with probable unequal variances [39] as would be the case with subgroups of teenagers in their framing of sexual behavior. We tested for significance of differences between group means at an overall alpha of .05, applying the Dunn-Bonferroni procedure to control for possible inflation of Type 1 error with multiple pair-wise comparisons.

## Results

We present the statement of findings regarding our research aims to determine : 1) the conceptual framing of HIV prevention by the Pentecostal teenagers; and 2) possible moderating effects of having had first sex, gender and age on the importance the church youths attached to specific HIV prevention strategies they endorsed. As illustration, we provide example statements for the content of the specific HIV prevention concepts as listed by the teenagers (see Table 3).

**Table 3:** Sample statements per concept cluster with means and standard deviations by sexual experience

**Item**	**Statement by cluster**	**First sex**		**Item**	**Statement by cluster**	**First sex**	
		**Yes**	**No**			**Yes**	**No**
	**Cluster 1. Biblical perspectives** (Items =9; α = .83)	Mean score and SD			**Cluster 4. Facts about HIV and AIDS** (Items = 9; α = .80)	Mean Score and SD	
43	Trust in God always	4.73 (.66)	4.77(.83)	5	Caring for those with HIV and AIDS	4.27( 1.08)	4.43(1.11)
38	Protecting and valuing your virginity	4.42(1.13)	4.73(.98)	12	Voluntary testing and counselling	4.04(1.27)	4.16(1.18)
22	Say no to sex before marriage	4.60(1.21)	4.63(1.15)	1	Sexually transmitted infections	4.19(1.29)	3.98(1.13)
50	Ask for partner from God	4.56(1.27)	4.55(1.34)	3	What is HIV and AIDS	3.81(1.12)	3.79(1.08)
	**Cluster mean and SD**	4.34(.32)	4.37(.42)		**Cluster mean and SD**	3.52(.47)	3.65(.39)
	**Cluster 2. Future focus** (Items = 11; α = .85)				**Cluster 4. Prevention education** (Items =11; α = .69)		
31	Importance of life targets and setting personal goals	4.35(1.07)	4.40(1.13)	39	Life consequences of unwanted pregnancies	3.40(1.25)	3.81(1.21)
19	Taking responsibility for one’s future	4.04(1.11)	4.38(1.15)	24	Risks from early involvement in love affairs	3.83(1.09)	3.68(1.17)
47	Aim for a career	4.15(1.08)	4.20(1.12)	30	Risks from sex for money	3.15(1.15)	3.65(1.28)
27	School first sex after	4.02(1.31)	4.18(1.18)	26	Proper use of contraceptives	3.15 (1.16)	3.29(1.27)
	Cluster mean and SD	3.91(.33)	4.06^*^(.23)		**Cluster mean and SD**	3.33 (.31)	3.33(.37)
	**Cluster 3. Community norms** (Items =10; α = .74)						
37	Talk about HIV with peers, teachers and parents	4.23(1.22)	4.28(1.03)				
10	Healthy living	4.37(1.02)	4.22(1.13)				
44	Choose friends wisely	3.94(1.17)	4.22(1.24)				
32	Self-pride and valuing yourself	3.88(1.21)	3.79(1.16)				
	**Cluster mean and SD**	3.68^*^(.47)	3.74(.47)				

^*^p < .01.

### Structure and salience of the HIV prevention concepts by the church teenagers

Figure 1 shows the cluster map of the conceptual framing of HIV prevention by the Pentecostal teenagers. The higher stacked clusters are those more prominently represented in the church youths’ construction of their HIV prevention concepts as compared to the lower stacked clusters.

The order of importance of the conceptual clusters for HIV prevention by the church youths was as follows: Biblical perspectives (Mean = 4.36; SD = .42), Future focus (Mean = 3.99; SD = .28); Community Norms (3.71; SD = .47), Facts about HIV and AIDS (Mean = 3.56; SD = .42) and Prevention Education (Mean =3.33; SD = .32) (see Table 3). Biblical perspectives refer to the belief that following religious teachings prevents one from contracting HIV. Future focus refers to the fact that having long-term life goals rather than seeking immediate gratifications prevents behaviors that could lead to contracting HIV. Community norms refer to knowledge and awareness of social standards in the general population, inclusive of those that influence sexual decisions. Facts about HIV and AIDS refer to scientific knowledge of the nature of HIV and AIDS, including transmission pathways. Prevention Education is life skills learning, including protection from contracting sexually transmitted infections.

Framing of HIV prevention from Biblical perspectives was most important to the church youths infection risk reduction than prevention from secular-oriented strategies such as Facts about HIV and AIDS, t (df = 246) = 15.53, p. < .001, and Prevention Education, t (df = 246) = 22.50, p < .001. Biblical perspectives were rated higher than Future Focus, t (df = 229) =8.45, p. < 0001 and Community norms, t (df = 260) = 11.89, p < 001. The church youths rated Biblical perspectives more salient to their HIV prevention regardless of sexual debut or age. The hypothesis that Pentecostal church youths conceptually frame HIV prevention based on faith-oriented teachings to primarily prevent them from contracting HIV was supported by the findings.

### Sexual experience effects on salience of secular-oriented HIV prevention concepts

Our analyses considered the relative salience of secular-oriented framing of HIV prevention components contrasted to each other. For these analysis, we included only those with non-missing data and reporting on their first sex (n =133). Table 4 presents the group difference mean scores on the importance of HIV prevention concept clusters with and without first sex. With four between-group HIV prevention cluster contrasts, the Dunn-Bonferroni alpha for significance is set at .01 (i.e., .05/4). Based on these analyses, youths with first sex rated Future focus relatively less important to their framing of HIV prevention (Mean =3.91, SD = .33), than did those with no first-sex (Mean =4.01; SD = .23) t (df = 151) = 3.46, p. < 001. They also considered Facts About HIV and AIDS relatively less important to their framing of HIV prevention (Mean =3.52, SD = .47) than peers with no first sex (Mean =3.65, SD = .39), t (df = 88) = 1.97, p. <. 01. Within the subgroup of youths with or without first sex, the secular-oriented framing of HIV prevention was less well differentiated. Thus, the hypothesis that first- sex moderated the relative salience to the church youths of secular-oriented framing of HIV risk reduction was supported by the findings.

**Table 4:** Mean importance difference scores among secular HIV prevention concept clusters by first sex (N = 133)

**Cluster mean (SD)**	**First sex (n = 47)**				**Cluster mean (SD)**	**No first sex (n = 86)**				**Between groups**
	**1.**	**2.**	**3.**	**4**		**1.**	**2.**	**3.**	**4**	
1. FHIV	.00				1. FHIV	.00				-.13
3.52 (.47)					3.65 (.39)					
2. PED	.19	.00			2. PED	.32^*^	.00			.00
3.33 (.31)					3.33 (.37)					
3. CNS	-.16	-.35^*^	.00		3. CNS	-.09	-.41	.00		-.06
3.68 (.47)					3.74 (.47)					
4. FFC	-39^*^	-.58^*^	-.23	.00	4. FFC	-.41^*^	-0.73	.32^*^	.00	-.15
3.91(.33)					4.06 (.23)					

Note. The numbers in brackets are standard deviations. FHIV = Facts about HIV and AIDS, PED = Prevention Education, CNS = Community Norms, FFC = Future Focus.

^*^p < .001.

### Gender and age effects of the salience of secular HIV prevention concepts

We examined the possible effects of gender and age as moderators of the perceived salience of secular-oriented framing of HIV prevention (see Table 5). The female youths placed relatively lower salience on the secular-oriented HIV prevention strategies of Prevention Education (Mean = 3.49, SD = .37) than the males (Mean =3.73, SD = .33), t (df =148) = -4.78, p. < .001. They also had a relatively lower regard of Future Focus as a HIV prevention strategy (Mean =3.79, SD = .37) than their male peers (Mean =4.02, SD .37), t (df = 133) = 4.25, p. < .001. There were no gender differences in the perceived salience of the secular-oriented HIV prevention strategy to learn of Facts about HIV and AIDS and influence of Community Norms. The hypothesis that gender would mediate the relative salience of secular-oriented framing of HIV prevention was partially supported by the findings.

**Table 5:** Mean importance difference scores among secular HIV prevention concept clusters by age (N = 213)

**Age group**										
	**Younger (< 18 years)**					**Older (> 18 years)**				**Between groups**
	**( n = 59)**					**(n = 154)**				
**Clusters mean (SD)**	**1.**	**2.**	**3.**	**4**	**Clusters mean (SD)**	**1.**	**2.**	**3.**	**4**	
1. FHIV	.00				1. FHIV	.00				-.41
3.31 (.52)					3.72 (.35)					
2. PED	-.16	.00			2. PED	.11	.00			-.14
3.47 (.48)					3.61 (.46)					
3. CNS	-.09	.07	.00		3. CNS	-.05	-.16	.00		-.37
3.40 (.60)					3.77 (.39)					
4. FFC	.31	-.45^*^	-.52^*^	.00	4. FFC	-.26	.61^*^	-.21^*^	.00	.06
3.92 (.41)					3.98 (.33)					

Note. The numbers in brackets are standard deviations. FHIV = Facts about HIV and AIDS, PED = Prevention Education, CNS = Community Norms, FFC = Future Focus.

^*^p < .001.

The older youths (18 years or older) rated Facts About HIV and AIDS (Mean =3.72, SD = .35) relatively more important than did the younger peers (Mean =3.31, SD = .52), t (df = 37) = p. < .001. The older youths also rated Community norms (Mean =3.77, SD = .39) relatively more important to their HIV prevention than did their younger peers (Mean =3.40, SD = .60), t (df = 36) =2.93 = p. <.01. There were no age group differences in the perceived relative salience of Prevention Education and Future Focus. The study findings are in partial support of the hypothesis that age of teenager would moderate the relative importance to the church youths of secular-oriented framing of HIV prevention.

## Discussion

The conceptual frameworks for HIV prevention held by Pentecostal church youths clustered into five groupings by priority: Biblical teachings, Future focus, Community Norms, Facts about HIV and AIDS and Prevention education. A unique finding from this study is the mapping of the ways church youth understood HIV prevention in the context of their religion and secular influences. This study yielded both the conceptual structure and content for a prospective HIV prevention intervention with church community youths from a developing country.

Although the youths conceptually framed their HIV prevention concepts to include both faith and secular-oriented concepts, they prioritized faith-oriented concepts relatively more than they did secular-oriented concepts. The finding is consistent with the view that the church as a knowledge environment implicitly essentializes faith-informed framing of health concepts [29,31]. Related studies have documented faith-oriented essentialization in response to the HIV pandemic by Pentecostal family type of churches in Botswana [21,24,29], and in Zimbabwe [15,16] and Mozambique [40]; -countries which share the same cultural outlook with Botswana. Thus, there appears to be an implicit understanding among the church youths that observance of the church’s core teachings about HIV prevention (e.g., sexual abstinence for unmarried youths) would provide robust protection against a cross-generational pandemic like HIV. However, this perspective might put at higher HIV risk those youths for which adoption of comprehensive secular HIV prevention intervention might be appropriate (e.g., the sexually active) [41].

Among the secular-oriented HIV prevention strategies, Future focus was the most highly prioritized for HIV risk prevention strategies. It could be regarded as both a faith and secular health protection strategy. For instance, church members would perceive a future willed by a higher spiritual authority, including their prospective health [42]. Future focus for church youths would also mean living church institutionally supported sexual health norms (e.g., abstinence only- until-marriage) and which in this case would reduce risk for HIV infection. As a secular health promotion concept, Future focus is tied to identifying and pursuing goals important to good health and the means to attain them [43]. This would include healthy use of leisure time, use of contraception, and avoiding situations that would expose one to risk for contracting HIV [44]. Future studies should examine the education processes to support choice of healthy futures by the Botswana Pentecostal church youths in a country with high HIV prevalence.

The salience of secular-oriented framing of HIV prevention by the church youths was moderated by sexual experience so that those with first sex considered Facts about HIV and AIDS as relatively less important to their HIV prevention as peers with no first sex. On the one hand, the seeming relative discounting of proven secular HIV prevention concepts like Facts about HIV and AIDS would suggest a higher risk for contracting HIV among the church youths with first sex. On the other hand, with adherence to the church’s framing of HIV prevention (e.g., abstinence for unmarried youths), church youths may perceive to achieve robust health protection with less ego resource depletion [45,46] than would be with multi-concept secular-oriented interventions (which would lower their risk for contracting HIV). Ego-depletion theory proposes that people seek to conserve their personal (ego) energy resource in their health maintenance by doing the minimum necessary to achieve desired health outcomes [45,47]. In transacting contrasting knowledge systems (faith versus secular), Pentecostal teenagers in their framing of HIV prevention concepts may align with church teachings, conserving their health protection energies and resulting in lower risk for contracting HIV. Future research should examine the relative explanatory value of ego-resource conservation and dissonance reduction as constructs for the framing of HIV prevention with the Pentecostal church youths.

The female church youths perceived secular-oriented HIV prevention concepts of Prevention Education and Future focus to be relatively less salient to their HIV prevention that did the male youths. This may reflect a cultural imbalance in how females and males perceive to be in control of their futures in Batswana culture. For instance, females in the patriarchal Botswana cultural context may perceive to hold less decisional powers about their futures, including their sexual and reproductive health [31]. This effect may persist even in the context of church, which also is mostly patriarchal in culture [2]. Thus, female Botswana Pentecostal teenagers are at elevated risk for HIV infection, partly from the socio-cultural inequities that constrain choices by females to direct their futures. Future studies could use qualitative inquiry approaches to explore the futures that Botswana female teenagers perceive to control or wish for and the ways by which these could be enabled for health promotion with them.

The older teenagers were more differentiating in their rating of the comparative worth of the secular-oriented HIV prevention concepts suggesting greater exposure to secular HIV prevention education with increases in age. This finding might be variously explained by exposure to the country’s formal education system. HIV prevention education is mandatory in Botswana schools [48], and the church teenagers would have been exposed to secular HIV prevention education formally through the school curriculum and also through community oriented public health education [31]. The older youths having progressed higher or further in the education system would likely perceive secular influences on their HIV prevention than would the younger peers with relatively less formal education. The church youths may have sexual partners who are from the secular community or with different sexual health attitudes different from those of their FBO, inclining them to privately frame their HIV prevention to align with their romantic or sexual partners. Church youths are part of the secular community, and HIV is mostly acquired from social networks. Future studies should examine social networks by church youths as conduits for HIV prevention information and education.

### Implications for HIV prevention education with church youths

Church youths perceived both faith and secular influences important to their HIV prevention. This means that some aspects of existing comprehensive, evidence informed HIV prevention interventions might be of service with Pentecostal church youths if tailored to be of value-add to church institutionally endorsed concepts [34,49]. An implication of the findings from this study is that while HIV prevention interventions with church community youths could be customized to the church knowledge environment, the church teenagers recognize and value heath protective secular oriented teachings. For instance, sexually active teenagers in this Pentecostal faith community for abstinence outside marriage (A) would likely look to secular concepts (possibly condom use: C) for their health protection than to church/faith concepts only, even with a higher regard for faith-oriented framing of HIV prevention. If church youths engaged in premarital sex from privately framing of their sexual decisions contrary to the church “A” teachings, while also undervaluing secular-oriented prevention concepts (e.g., knowledge about HIV and AIDS, use of condoms), then their HIV risk would increase.

Although the relative openness to secular-oriented framing of HIV prevention by the church youths represents an exciting opportunity for public health, an effective collaboration with the faith sector will require a better understanding of their specific faith traditions; - to better align HIV prevention messages and identify resources for sexual health education. For instance, while church communities may share a preference for faith-oriented framing of HIV prevention, there may be shades of differences in perceived importance of the specific practices underpinned by the same generic HIV prevention concept (e.g., Biblical perspective). For instance, churches for abstinence only-until-marriage impose a more restrictive sexual health norm standard on their youths congregates that those prioritizing abstinence but with valuing of secondary abstinence for those who may have indulged while unmarried [50]. These differences in faith-oriented concepts emphasis may arise from the cultural-historical traditions of specific faith traditions as interpreted by the church leadership in the context of current health issues [15,40]. Thus, faith traditions may have diversity in the content of their specific HIV prevention concepts and which would be important for health promotion partnerships with them.

### Limitations of the study

First, the study investigated the conceptual framing of HIV prevention by youth members of a prosperity oriented Pentecostal church, and their priority concepts may be different from those of faith communities with a different ideology. For instance, youth members of a faith community with a vengeful view of God (33, 34) may be less accepting of secular-oriented HIV prevention. In this regard, a study on the constructions of HIV prevention concepts by the church leadership would clarify the likely influences of church ideology on the ways in which the Pentecostal church youths framed their HIV prevention. A substantial overlap between the conceptual framing of HIV prevention between the church youths and leadership would suggest higher prospects for the adoption implementation of a prevention intervention to result.

Second, perceptions of HIV prevention concepts rather than the actual HIV prevention behaviors the church youths engaged were studied. Evidence is needed on context and types of sexual decisions the church youths engage to protect themselves from contracting HIV. Some of the church youths may see no disconnect between their being sexually active while in a church officially endorsing abstinence, and if they privately framed their sexual decisions to be consistent with the church’s compassionate view of God.

Third, the relatively higher representation of female youths congregates compared to males may have biased in unknown ways the structure and salience of HIV prevention concept map observed. Future studies could use a larger enrollment of the male youths with confirmatory tests [51] to check on the comparability of HIV prevention concept maps by gender.

Fourth, the study did not seek to explore the framing of HIV prevention by church youths already infected from any cause. The incidence of new infections in the church membership will continue to increase in the absence of a cure as is the case in the general population. Infected church members will need FBO pastoral and treatment care support, among others socially networked resources, to prevent secondary infection and transmitting the HIV to others [5]. Future studies should consider the framing of HIV prevention by FBOs with youth members living with HIV and AIDS.

Fifth, despite privacy protections for the data reporting, some of the youths may have underreported on their sexual debut for social desirability effects, and particularly since the data were collected at the congregation or church sites rather a neutral community center. Social desirability effects to not disclose own sexual activity may have resulted in the relatively lower numbers of youths self-identifying as with first sex experience, which would under-power the related analysis. Future studies should engage church youths for study in neutral venues such as community centers where the youths may feel less constrained in expressing their views by the context of study.

## Conclusion

Botswana Pentecostal church youths perceive a structure of concepts to prevent them from contracting HIV in which faith teachings are the most important, and although they also recognize secular-oriented prevention concept. Homogenizing church knowledge environments effects may explain the strong prioritization by the Pentecostal youths of faith-oriented HIV concepts compared to secular-oriented concepts.

Seemingly, the church youths may have lower risk for HIV if with adherence to church teachings. However, the church knowledge environment if it essentialzed faith-supported HIV prevention concepts only may deny some youth congregates the sexual health protections possible with comprehensive or inclusive public health concepts. Pentecostal church youths also value secular HIV prevention concepts, suggesting that they would be receptive to secular-oriented interventions. Female church youths prospectively carry a higher risk for HIV infection from gender oriented socio-cultural inequities negating control of their futures. HIV prevention interventions with Botswana Pentecostal church youths should be designed to address gender based vulnerability of the female teenagers. Church youths likely would align their HIV prevention concepts to those supported by their FBO, and education support on secular oriented prevention concepts would be helpful to the older and sexually active teenagers.

## Competing interests

The authors have no competing interests.

## Authors' contributions

EM conceptualized and directed all aspects of the study including the manuscript writing. FN was in-country project director, coordinated the data acquisition and participated in writing the introduction and discussion of the findings. JM participated in the data acquisition and church community partner engagement and also the writing of the introduction. LT participated in the data acquisition and writing of the discussion and interpretation of findings. JBB helped the manuscript writing and specifically clarification of the goals, the description and discussion of the findings. All authors read and approved the final manuscript.

## Pre-publication history

The pre-publication history for this paper can be accessed here:

http://www.biomedcentral.com/1471-2458/14/225/prepub
